# *Drosophila cbl* Is Essential for Control of Cell Death and Cell Differentiation during Eye Development

**DOI:** 10.1371/journal.pone.0001447

**Published:** 2008-01-16

**Authors:** Yuan Wang, Christian Werz, Dongbin Xu, Zhihong Chen, Ying Li, Ernst Hafen, Andreas Bergmann

**Affiliations:** 1 Department of Biochemistry and Molecular Biology, The University of Texas, M.D. Anderson Cancer Center, Houston, Texas, United States of America; 2 Institute of Molecular Systems Biology, Eidgenössische Technische Hochschule Zürich, Zürich, Switzerland; Baylor College of Medicine, United States of America

## Abstract

**Background:**

Activation of cell surface receptors transduces extracellular signals into cellular responses such as proliferation, differentiation and survival. However, as important as the activation of these receptors is their appropriate spatial and temporal down-regulation for normal development and tissue homeostasis. The Cbl family of E3-ubiquitin ligases plays a major role for the ligand-dependent inactivation of receptor tyrosine kinases (RTKs), most notably the Epidermal Growth Factor Receptor (EGFR) through ubiquitin-mediated endocytosis and lysosomal degradation.

**Methodology/Principal Findings:**

Here, we report the mutant phenotypes of *Drosophila cbl* (*D-cbl*) during eye development. *D-cbl* mutants display overgrowth, inhibition of apoptosis, differentiation defects and increased ommatidial spacing. Using genetic interaction and molecular markers, we show that most of these phenotypes are caused by increased activity of the *Drosophila* EGFR. Our genetic data also indicate a critical role of ubiquitination for *D-cbl* function, consistent with biochemical models.

**Conclusions/Significance:**

These data may provide a mechanistic model for the understanding of the oncogenic activity of mammalian *cbl* genes.

## Introduction

Normal cellular function and tissue homeostasis is dependent on the precise regulation of several signal transduction pathways that control cell proliferation, cell differentiation and cell survival. Each cell integrates an array of extracellular signals into appropriate cellular responses. Deregulation of these processes causes developmental abnormalities and human diseases including cancer. However, we still lack a clear understanding of how these processes are integrated in the context of a developing organism.

The development of the retina in the *Drosophila* compound eye has long been a model system to study how extra-cellular signaling generates precise cellular differentiation patterns (reviewed by reference [1]). The compound eye is composed of ∼800 ommatidia, repetitive units each containing a precise number of different cell types. The adult fly eye develops from a monolayer epithelium—the eye imaginal disc. In early larval stages cells in the eye imaginal disc proliferate to provide the cellular mass for eye development. During mid-third instar larval stage, cellular differentiation starts at the posterior end of the eye imaginal disc, which coincides with formation of the morphogenetic furrow (MF) that sweeps across the disc from posterior to anterior [1], [2]. As the MF progresses towards the anterior, cells located behind the MF start differentiating into distinct cell types in a strict sequence to form the ommatidium. Each ommatidium has eight photoreceptor neurons or “R” cells (R1–R8). R8 is the first R cell to be specified, and serves as the founder cell for recruitment of the other R cells in the order R2/R5→R3/R4→R1/R6→R7, followed by four non-neuronal cone cells during late third instar larval stage, and three classes of pigment cells during early pupal stages [3]. Finally, after specification of these cell types has been completed, all surplus undifferentiated cells are removed by apoptosis [3], [4]. This occurs between 26–30 hours after puparium formation [5].

The specification of cell fate in the developing *Drosophila* retina is controlled by combinatorial signaling. Two receptor tyrosine kinases (RTKs), the epidermal growth factor receptor (EGFR) and Sevenless (Sev), contribute to retinal development [6], [7]. Activation of EGFR by the secreted ligand Spitz (sSpi), a transforming growth factor (TGF-α) homologue, regulates the specification of all R cells in the developing eye, except R8 [6], [8], [9]. Over-expression of sSpi causes an over-recruitment of all cell types, while expression of dominant negative *EGFR* (*EGFR^DN^*), or shifting a temperature-sensitive *EGFR* allele to the non-permissive temperature leads to an impairment of differentiation [6], [10], [11]. Ommatidia mutant for *argos*, *gap1* and *sprouty*, three negative regulators of EGFR, contain extra R and cone cells surrounded by more secondary and tertiary pigment cells in the lattice [12]–[18]. In addition, EGFR signaling is utilized for cell survival during *Drosophila* eye development, due to its negative regulation of *hid*, a cell death-inducing gene [19]–[21]. In contrast to the EGFR which controls the development of all R cells in the ommatidium except R8, *sev* is required only for R7 differentiation [22].

As important as the activation of cell surface receptors is their inactivation for appropriate control of cell number and differentiation. The proto-oncogene Casitas B-lineage lymphoma (Cbl) was first identified as a retroviral transforming gene product that induces pre-B cell lymphomas and myeloid leukemia [23]. Cbl is involved in many signaling events through its function as a multi-domain adaptor protein and has been best characterized as a negative regulator of RTKs, mostly EGFR (reviewed by [24], [25]). This concept grew out of genetic studies performed in *C.elegans* in which Sli-1, the Cbl ortholog, attenuates the activity of Let-23, the EGFR equivalent, in vulval development. [26]. Mammals contain three Cbl genes known as c-Cbl, Cbl-b and Cbl-3, which function as negative regulators of EGFR [25], [27], [28]. Knock-out mice of *c-cbl*, *cbl-b* and *cbl-3* have no obvious developmental phenotypes except in the immune system suggesting that they are functionally redundant [29]–[32]. *Drosophila* has only one *cbl* gene, referred to as *D-cbl* [33]–[35], eliminating the problem of redundancy, and the genetic characterization of *D-cbl* mutants may reveal more information about its oncogenic role. For example, an isoform of *D-cbl,* which mimicked the oncogenic viral *cbl* (*v-cbl*), demonstrated that *v-cbl* acts in a dominant negative manner [35]. Furthermore, consistent with studies in *C.elegans* and mammalian cell culture, *D-Cbl* has been shown to function as a negative regulator of EGFR during dorsoventral patterning in oogenesis and guided migration of border cells [36], [37]. A loss-of-function analysis of *D-cbl* for eye development in *Drosophila* has not been reported.

**Figure 1:**
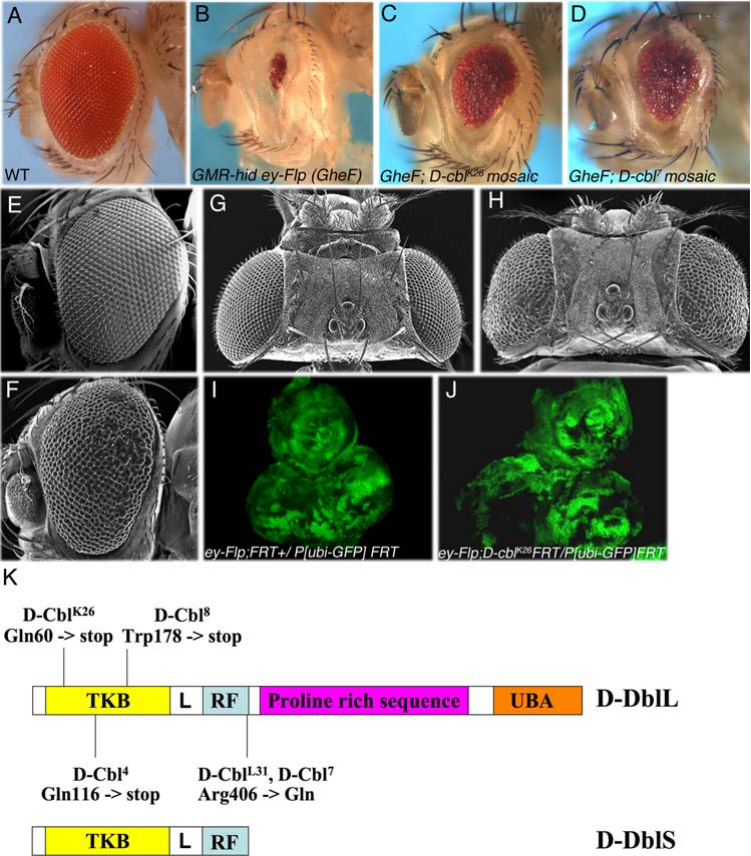
Identification and characterization of *D*-*cbl* mutants. (A) Wild-type (WT) fly showing normal eye phenotype. (B) *GMR-hid ey-FLP (GheF)* small eye phenotype. (C,D) *GheF*;*D-cbl^K26^* and *GheF*;*D-cbl^7^* mosaics significantly suppress the *GMR-hid* small eye phenotype. Genotype: *GheF*; *D-cbl FRT80*/*P[w^+^] FRT80* (E,G) Scanning electron micrograph (SEM) of wild-type adult eye (E) and head (G). (F,H) *ey*-*FLP*/Minute-induced mosaics of *D-cbl^K26^* display rough eyes (F) and enlarged heads (H). Genotype: *ey-FLP*; *D-cbl^K26^ FRT80*/*M(3)i55 FRT80.* (I,J) Eye-antennal discs of 3^rd^ instar larvae of *D-cbl^K26^* mosaics (I) are larger compared to wild-type (J). Scale bar 20um. (K) Domain structure of long (L) and short (S) isoforms of D-cbl. The relative locations of three non-sense mutations and two missense mutations in D-Cbl are indicated. *D-cbl^L31^* and *D-cbl^7^* affect the same residue in the RING domain. TKB-Tyrosine Kinase binding domain; L–Linker; RF-RING finger; UBA-ubiquitin-associated domain.

Mechanistically, Cbl binds tyrosine-phosphorylated EGFR through its tyrosine kinase binding (TKB) domain [38] (see also **Fig. 1K**). The E3 ligase activity of the RING domain of Cbl directs the mono-ubiquitination of activated EGFR at multiple sites, which promotes endocytosis and endosomal sorting for lysosomal degradation of the receptors [39]–[43]. *D-cbl* encodes two alternatively spliced isoforms, D-cblSHORT (D-cblS) and D-cblLONG (D-cblL), both of which contain the TBK and the RING E3 ubiquitin ligase domains, while D-CblL also has proline-rich (SH3 binding) and UBA domains similar to c-Cbl and Cbl-b [33]–[35] (see **Fig. 1K**).

Here, we present the characterization of the *D-cbl* mutant phenotype for eye development. *D-cbl* mutants display overgrowth and lack of developmental apoptosis. Mutant ommatidia contain increased numbers of photoreceptors (mostly R7), cone and pigment cells. Genetic interaction tests indicate that *D-cbl* regulates the EGFR pathway during eye development consistent with its proposed role as negative regulator of EGFR. Our genetic data indicate a critical role of ubiquitination for D-cbl function, in accord with biochemical models. In summary, these data provide a genetic model for the understanding of the oncogenic activity of mammalian *cbl* genes.

## Results

### Isolation and characterization of *D-cbl* mutants

In a mutagenesis screen, we isolated five mutant *D-cbl* alleles as recessive suppressors of the small eye phenotype caused by expression of the pro-apoptotic gene *hid* under control of the eye-specific *GMR* enhancer (*GMR*-*hid*; **Fig. 1A–D**). For details about the *GMR*-*hid* suppressor screen see Material and Methods, and references [44]–[46]. Because D-Cbl is a known negative regulator of EGFR, and because increased EGFR activity inhibits the pro-apoptotic function of Hid [19]–[21], the isolation of *D-cbl* mutants as suppressors of *GMR*-*hid* can be explained by its effect on EGFR. However, what sparked our interest in characterizing *D-cbl* for eye development are the mutant phenotypes without *GMR*-*hid* expression. The eyes appear rough and bulgy with larger ommatidia (**Fig. 1E, F**), and *D-cbl* mutant heads are overgrown (**Fig. 1G, H**). The overgrowth phenotype is already visible in eye-antennal imaginal discs of 3^rd^ instar larvae (**Fig. 1I, J**). Both, the strong rough eye and the overgrowth phenotype cannot be solely explained for by inhibition of apoptosis. Thus, we characterized the *D-cbl* mutant phenotype during eye development in more detail.

DNA sequencing revealed missense and non-sense mutations (**Fig. 1K**). *D-cbl^7^* and *D-cbl^L31^* affect the same residue, the highly conserved Arg406 residue in the RING domain. The remaining alleles, *D-cbl^K26^*, *D-cbl^4^* and *D-cbl^8^* introduce premature STOP codons at positions 60, 116 and 178, respectively (**Fig. 1K**). *D-cbl^4^* is identical to a previously isolated allele, *D-cbl^F165^* [36]. At least *D-cbl^K26^* can be considered a null allele of *D-cbl*. Interestingly, all isolated alleles affect both the large and the small isoform of *D-cbl*. We did not recover mutant alleles that affect only the large isoform. All experiments in this study were performed with at least two alleles, the null allele *D-cbl^K26^* and the RING domain mutant *D-cbl^7^*, both of which show identical results.

### D-Cbl regulates the EGFR pathway in the *Drosophila* eye

**Figure 2:**
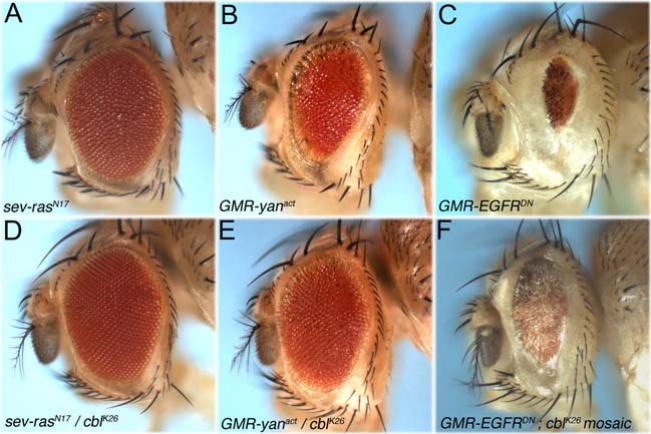
Genetic interaction between *D-cbl* and the EGFR pathway. (A,D) The rough eye caused by over-expression of dominant negative *ras^N17^* under the *sevenless* promoter (*sev-ras^N17^*) (A) is considerably suppressed when heterozygous for *D-cbl^K26^* (D). Genotype in (D): *sev-ras^N17^*; *D-cbl^K26^*/+. (B,E) The small eye phenotype caused by *sevenless*-induced expression of activated *yan* (*sev-yan^act^*) (B) is dominantly suppressed by heterozygosity for *D-cbl^K26^* (E). Genotype in (E): *sev-yan^act^* ; *D-cbl^K26^*/+. (C,F) Overexpression of *EGFR^DN^* under the control of the *GMR* enhancer (*GMR*- *EGFR^DN^*) causes a small eye (C). Genotype: *GMR-Gal4 UAS-EGFR^DN^*. *GMR-EGFR^DN^* is recessively suppressed in *D-cbl^K26^* mosaics (F). Genotype: *ey-Flp*; *GMR-Gal4 UAS-EGFR^DN^*; *D-cbl^K26^ FRT80/P[ubi-GFP] FRT80*.

D-Cbl has previously been shown to be a negative regulator of EGFR signaling during dorsoventral patterning in oogenesis and border cell migration [36], [37]. A similar loss-of-function analysis of D-cbl has not been done for eye development. We have performed several genetic interaction tests to determine whether D-Cbl controls EGFR signaling during eye development. First, heterozygosity of *D-cbl* considerably rescued the rough eye phenotype caused by over-expression of dominant negative *ras^N17^* (**Fig. 2A, D**). Second, *D*-*cbl^K26^* dominantly suppresses the eye phenotype caused by mis-expression of the active form of the repressor *yan* (*yan^act^*) (**Fig. 2B, E**), a target gene negatively regulated by EGFR signaling [47], [48]. Third, to more directly assess a role of *D-cbl* for the regulation of EGFR, we analyzed the effect of *D-cbl* mutants on the small eye phenotype caused by expression of a dominant negative allele of *EGFR*, *EGFR^DN^* [6], under control of the eye-specific enhancer *GMR* (*GMR*-*EGFR^DN^*) (**Fig. 2C**). EGFR^DN^ lacks the intracellular tyrosine kinase domain, but leaves the transmembrane and extracellular domains intact [6]. EGFR^DN^ is able to dimerize with endogenous EGFR, but trans-phosphorylation upon ligand binding does not take place and thus the dimer is unable to signal. However, the inhibition of endogenous EGFR by EGFR^DN^ is not complete as some R cells still survive and differentiate [6] (data not shown), which is not observed in strong *EGFR* mutant clones [49]. Thus, the small eye phenotype of *GMR*-*EGFR^DN^* is caused by partial inhibition of endogenous EGFR. In *D*-*cbl^K26^* mutant clones, the *GMR*-*EGFR^DN^* phenotype is moderately strongly suppressed (**Fig. 2F**) implying that loss of *D-cbl* partially restores the activity of endogenous EGFR inhibited by EGFR^DN^. Combined, these data suggest that *D*-*cbl* mutants contain increased EGFR activity suggesting that D-cbl negatively controls EGFR activity.

**Figure 3:**
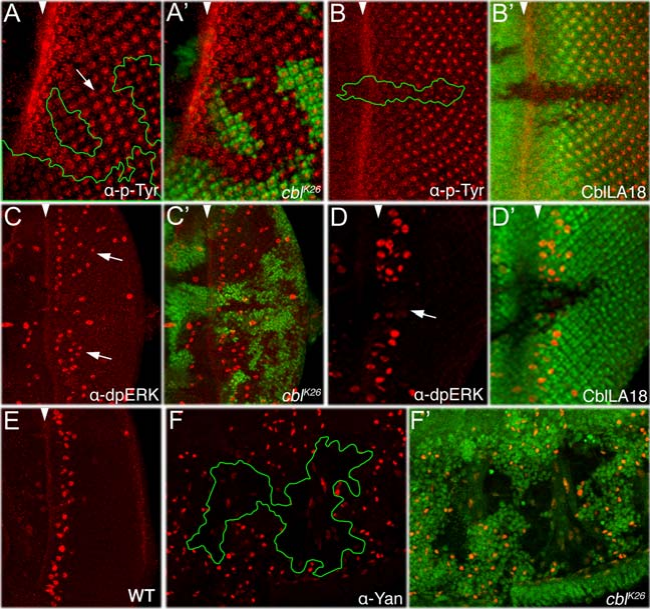
*D-cbl* regulates EGFR pathway activity. In panels (A–E), posterior is to the right. The morphogenetic furrow is marked by a white arrowhead. *D-cbl* clones in (A,C,F) and *D-cbl* overexpressing clones in (B,D) are marked by the absence of GFP. Genotype in (A,C,F): *ey-Flp*; *D-cbl^K26^ FRT80*/*P[ubi-GFP] FRT80*; genotype in (B,D): *hs-Flp*; *tub>GFP>Gal4*/*UAS-D-cblLA18* (> = FRT); genotype in (E): wild-type. (A,A') p-Tyr labeling is increased in *D-cbl* clones. (B,B') Overexpression of *D-cbl* suppresses p-Tyr labeling. (C,C') dp-ERK labeling persists in *D-cbl* mutant clones beyond the normal labeling immediately posterior to the MF (see arrows, compare to (E)). (D,D') Overexpression of *D-cbl* suppresses dp-ERK labeling in third instar larval eye discs. (E) dpERK labeling in third instar wild-type eye imaginal discs is restricted to one ommatidial column posterior to the MF. (F,F') Yan protein is reduced in *D-cbl* mutant clones in 35 hours APF eye discs.

To further confirm this notion we tested three molecular markers in loss-of-function and gain-of-function analyses of *D-cbl*. First, phospho-tyrosine labeling (p-Tyr) as a marker of RTK activity is increased in *D-cbl* clones in third instar larval eye discs (**Fig. 3A**). This is well visible in *D-cbl* clones crossing the MF and posterior to the MF (**Fig. 3A**). In the reverse experiment, overexpression of *D-cbl*, p-Tyr labeling is significantly reduced (**Fig. 3B**). Because EGFR is the only known RTK acting posterior to the MF in eye development (except Sev which we can exclude as a target of *D-cbl*, see below), the increased p-Tyr labeling is mainly caused by increased EGFR activity.

Second, in wild-type third instar larval eye discs, immunolabeling with dpERK, an antibody that recognizes activated MAPK acting downstream of EGFR, is detectable in one ommatidial column immediately posterior to the MF [50] **(** **Fig. 3E**). Further posteriorly, dpERK is not detectable suggestive of MAPK inactivation. In *D-cbl* mutant clones, dpERK labeling persists further posteriorly to the MF (**Fig. 3C**) suggesting lack of MAPK inactivation. Furthermore, the reverse experiment, overexpression of *D-cbl*, results in loss of dpERK labeling (**Fig. 3D**).

The third molecular marker used is Yan, a transcriptional repressor in the nucleus. In response to EGFR signaling, *yan* transcription is inhibited and Yan protein is proteolytically degraded [47], [48]. In pupal eye discs 35 hours after puparium formation (APF) Yan protein is strongly reduced in *D-cbl* clones (**Fig. 3F**) suggesting that they contain increased EGFR activity. Taken together, these data suggest that *D-cbl* negatively regulates EGFR signaling during eye development.

### *D-cbl* mutants block apoptosis and cause over-recruitment of all cell types in the eye

**Figure 4:**
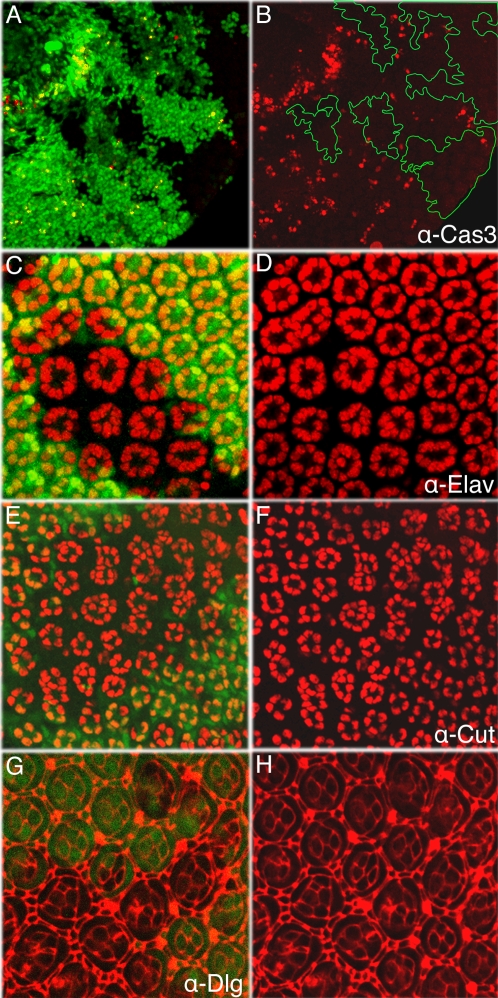
Over-recruitment of all cell types in *D-cbl* mutant ommatidia. Genotype in all panels: *ey-Flp*; *D-cbl^K26^ FRT80*/*P[ubi-GFP] FRT80*. (A,B) Cell death detected by cleaved (activated) caspase-3 (Cas3) staining is significantly reduced in D-*cbl^K26^* mutant clones in 28 hours APF eye discs. Outlines of some clones are shown in (B). (C–F) Anti-Elav staining for photoreceptor cells (C,D) and anti-Cut staining for cone cells (E,F) in 42 hrs APF pupal discs. 11.45 R cells and up to eight cone cells are visible in *D-cbl^K26^* ommatidia. (G–H) Anti-Dlg labeling of 42 hrs APF pupal discs visualizes the outline of cells and allows determining the number of pigment cells in D-*cbl^K26^* mutant clones of pupal eye imaginal discs.

In the *Drosophila* eye, EGFR signaling is utilized for cell survival and cell differentiation. Because *D*-*cbl* mutants cause increased EGFR signaling, we tested whether this has consequences for cell survival and cell differentiation in the fly eye. EGFR function is anti-apoptotic due to its negative regulation of *hid* [19]–[21]. Developmental cell death during eye development is maximal between 26 and 30 hours after puparium formation (APF) when surplus, undifferentiated cells are eliminated [5]. This elimination requires the pro-apoptotic function of *hid* [20], [21], [51]. To determine whether *D*-*cbl* mutants affect cell death, we labeled 28 hours APF eye discs with an antibody that recognizes cleaved and thus activated Caspase-3 (Cas3). In *D*-*cbl^K26^* mutant clones, developmental cell death is significantly blocked (**Fig. 4A, B**). This finding is consistent with the isolation of *D*-*cbl* mutants as suppressors of *GMR*-*hid* (**Fig. 1C, D**).

Next, we tested whether *D*-*cbl* mutant clones display differentiation defects. The eye disc is fully differentiated by 42 hrs APF [3]. Labeling of 42 hrs APF eye discs with Elav antibody, a R cell marker, revealed that each *D-cbl* mutant ommatidium contains on average 11.45±1.03 (n = 35) R cells compared to eight in wild-type ommatidia (**Fig. 4C, D**). In addition, *D*-*cbl* mutant ommatidia contain up to eight cone cells compared to four in wild-type ommatidia (**Fig. 4E, F**) and about three primary pigment cells instead of two in wild-type (**Fig. 4G, H**). The total numbers of secondary and tertiary pigment cells has increased from nine in wild-type to an average of fourteen per *D-cbl* ommatidium (**Fig. 4G, H**). In summary, these data show that *D*-*cbl* mutants cause increased cellular survival and over-recruitment of all cell types disrupting the regular array of the ommatidia in the developing *Drosophila* eye.

### *D-cbl* specifically affects R7 development

**Figure 5:**
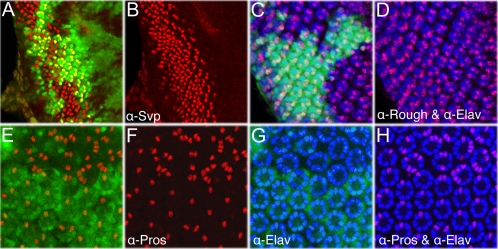
*D-cbl* affects R7 specification. Genotype in all panels: *ey-Flp*; *D-cbl^K26^ FRT80*/*P[ubi-GFP] FRT80*. (A,B) R3,4 and R1,6 are detected using anti-Svp antibody (red). (C,D) Expression of Rough (red) in R2,5,3,4 cells and Elav (blue) in all R cells. In *D-cbl^K26^* clones, R1-R6 cells are normal in number. (E–H) Specific increase of R7 cells as indicated by double labeling with anti-Prospero (Pros) (red) and anti-Elav (blue) antibodies.

We determined whether the over-recruitment of R cells in *D-cbl* clones affects all photoreceptors randomly, or specific types of photoreceptors. Labeling with antibodies directed against Rough (R2,5,3,4-specific) and Seven-up (Svp, R3,4,1,6-specific) did not reveal significant differences between *D*-*cbl* and wild-type tissue (**Fig. 5A–D**). However, labeling with an antibody against Prospero (Pros), a R7 marker, reveals 4.5±0.53 (n = 35) cells per *D*-*cbl* mutant ommatidium whereas in wild-type only one cell is detected (**Fig. 5E, F**). To determine whether the additional Pros-positive cells have photoreceptor character, we performed Pros and Elav double labelings. In *D*-*cbl* mutant clones, all Pros-positive cells are Elav-positive, suggesting that most, if not all, additional photoreceptors in *D*-*cbl* ommatidia are R7 cells (**Fig. 5G, H**). Thus, among the R cells, *D*-*cbl* specifically affects the specification of R7. A similar and specific increase in the number of R7 cells has been observed in eye tissue mutant for other negative regulators of EGFR signaling including *argos*, *gap1*, and *sprouty* [12], [16]–[18]. Thus, the over-recruitment phenotype in *D-cbl* mutants is typical for increased EGFR activity.

### Loss of *D-cbl* can compensate for loss of *sevenless*

In addition to EGFR, Sevenless (Sev), a second RTK, is involved in eye development. *sev* is only required for R7 specification and consequently *sev* mutants do not contain R7 cells [22]. However, expression of dominant active *sev* constructs gives rise to the recruitment of multiple R7 cells [52], similar to the *D-cbl* phenotype. Thus, it is formally possible that D-cbl also regulates Sev.

**Figure 6:**
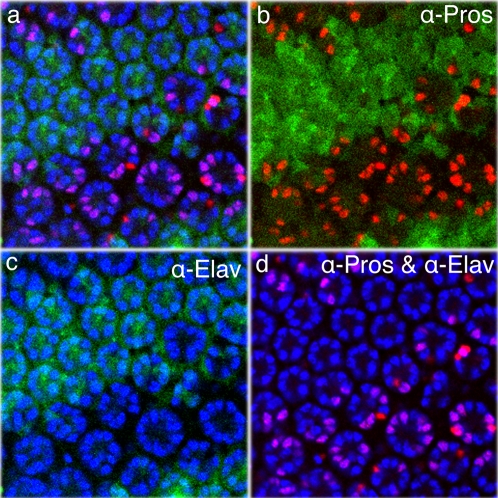
Loss of *D-cbl* compensates for loss of *sevenless*. The entire disc is mutant for *sev^d2^*. Homozygous *D-cbl* clones are marked by the absence of GFP. Anti-Pros (red) labels R7 cells, and anti-Elav (blue) labels all R cells. The R7 cells are absent in *sev^d2^* ommatidia, while 4.12±0.74 (n = 35) R7 cells are present in *sev^d2^;D-cbl^K26^* double mutant ommatidia. Genotype: *sev^d2^*/Y; *ey*-*FLP*/+ ; *FRT80 D-cbl^K26^*/*FRT80* P[*ubi*-*GFP*].

We tested this possibility. If the additional R7 cells in *D-cbl* clones result from hyper-activity of Sev, then these cells should require *sev* for their specification. However, genetic removal of *sev* using the null allele *sev^d2^* [53] has no effect on R7 specification in *D-cbl^K26^* mosaic background **(** **Fig. 6**). The average number of total R cells in *sev^d2^*, *D-cbl^K26^* mutant ommatidia is 11.12±0.74 (n = 35), containing more than four R7 cells per ommatidium (**Fig. 6**). The GFP-positive area is single mutant for *sev^d2^* and hence does not form R7 (**Fig. 6B, D**). Thus, in *D-cbl^K26^* mutants, the R7 cells can complete their differentiation program even in the absence of Sev, suggesting that the increase of EGFR signaling in *D-cbl^K26^* can compensate for loss of Sev. A similar *sev*-independent mode of R7 specification has been observed for other negative regulators of the EGFR pathway such as *gap1* and *sprouty* [16]-[18].

## Discussion

The phenotypic characterization of *D-cbl* mutants for eye development in *Drosophila* allows making four important conclusions: First, *D-cbl* plays an important role for the negative regulation of EGFR activity. Second, loss of *D-cbl* causes severe mis-specification and over-recruitment defects. Third, loss of *D-cbl* blocks developmental apoptosis. Fourth, loss of *D-cbl* causes tissue overgrowth at the organismal level, providing a model to study the oncogenic activity of mammalian *cbl* genes.

### D-Cbl negatively regulates EGFR

*D-cbl* mutant ommatidia contain increased numbers of R7, cone and pigment cells. Similar phenotypes have been observed in mutants of other negative regulators of the EGFR pathway such as *gap1*, *argos* and *sprouty* [12], [16]-[18]. We also confirmed a regulatory role of *D-cbl* for EGFR activity in genetic interaction studies (**Fig. 2**). Furthermore, using the molecular markers p-Tyr, dpERK and Yan as readouts for EGFR activity we showed that *D-cbl* clones contain increased RTK and MAPK activity. Likewise, overexpression of D-cbl blocks p-Tyr and dpERK labeling. Thus, the *D-cbl* mutant phenotypes in the eye are consistent with increased EGFR activity and suggest that D-Cbl negatively regulates EGFR, in accord with previous reports [34], [36], [37].

In contrast to *argos*, *gap1* and *sprouty*, *D-cbl* does not appear to regulate all RTKs. For example, D-cbl does not influence Torso [36], a RTK involved in specification of the termini in the *Drosophila* embryo [54]. Here, we have demonstrated that D-cbl does not control the Sev RTK. This difference likely reflects the direct mode of EGFR regulation by D-cbl, while *argos*, *gap1* and *sprouty* act downstream in the Ras/MAPK pathway which is shared by all RTKs. Biochemical data has demonstrated that mammalian Cbl proteins directly bind to tyrosine-phosphorylated EGFR and ubiquitylates it for endocytosis and lysosomal degradation [38]–[43]. Although we have not verified a similar biochemical mechanism for the interaction between *Drosophila* EGFR and D-Cbl, it is likely that the mechanism is similar. This notion is supported by the isolation of two *D-cbl* alleles affecting the RING domain (**Fig. 1K**). The RING domain contains an E3 ubiquitin ligase activity which targets the EGFR for ubiquitylation [39]. The mutant phenotype of *D-cbl^7^* affecting the RING domain is indistinguishable from the null allele *D-cbl^K26^* (data not shown), further supporting an essential role of ubiquitylation for D-cbl function.

It is unclear why only the number of R7 cells is affected whereas the remaining R cells are normal in number although R1–R6 also require the EGFR for specification. However, it suggests that the sequence of events during R cell specification is normal in *D-cbl* clones. The fact that *D-cbl* clones contain up to four additional R7 cells is likely due to the fact that R7 and the four cone cells are developmentally equivalent. These five cells express *sev* and all have the capacity to become R7 if Sev or downstream components are activated [52]. Thus, the additional R7 cells in *D-cbl* clones likely represent transformed cone cells.

However, this transformation does not mean that the cone cells are lost in *D-cbl* clones. In contrast, we even observe an over-recruitment of cone cells. Interestingly, the cone cell over-recruitment in *D-cbl* mutants does not occur during pupal stages as suggested for *gap1* [16]. It occurs at the correct developmental time in late third instar eye development (data not shown). Thus, the over-recruitment of several different cell types in *D-cbl* clones follows the same rules of reiterative use of the EGFR as compared to wild-type.

### EGFR-independent phenotypes of *D-cbl*

Despite the fact that the *D-cbl* mutant phenotypes are similar to the ones described for *argos*, *gap1* and *sprouty*, we noticed at least two phenotypes which appear to be specific for *D-cbl*. First, *D-cbl* mutant heads and imaginal discs are overgrown (**Fig. 1**). Second, the spacing between the ommatidial clusters is increased (see examples in **Fig. 4D, 4F**, and **6D**). Similar phenotypes have not been observed for *gap1*, *argos* and *sprouty* [12]–[18] (data not shown). It is unclear how these phenotypes are caused, but they may be independent of EGFR. Further studies are needed to clarify these observations.

### Implications for mammalian Cbl and oncogenesis

This work may also have some important implications for our understanding of the oncogenic nature of mammalian *cbl* [55]. Increased proliferation and reduced apoptosis are hallmarks of cancer [56]. *v-cbl* is a retroviral transforming oncogene causing pre-B lymphoma and myeloid leukemia [23]. *v-cbl* contains only the TKB domain [24] and behaves genetically as a dominant negative mutant [35]. Furthermore, inappropriate activation of mammalian EGFR can lead to various forms of human cancers [57]–[60]. Thus, genetic studies in model organisms may contribute to our understanding of oncogenic processes in mammals.

## Materials and Methods

### Identification of *D-cbl* mutant alleles

Eye-specific expression of *hid* under *GMR* enhancer control (*GMR*-*hid*) results in an eye ablation phenotype (**Fig. 1B**). Using the *GMR*-*hid ey*-*FLP* (*Gh*e*F*) method [44], we conducted an EMS-mutagenesis screen for chromosome arm 3L to identify recessive suppressors of the *GMR*-*hid* eye ablation phenotype. This method induces homozygous mutant clones in the eye by *ey*-*FLP*/*FRT*-mediated mitotic recombination in otherwise heterozygous background [61]. For *GheF* screening, *ey*-*FLP*; *FRT80* males were incubated with 25 mM EMS in 5% sucrose solution for 24 hours. After recovery for 3 hours, they were mated to *GheF*; *FRT80 P[w^+^]* females and incubated at 25°C. 45,000 F1 progeny were screened for suppression of the *GMR*-*hid* small eye phenotype. In the screen for 3L, 4 *dronc* [44] and 5 *D-cbl* alleles (this study) were recovered.

### *Drosophila* genetics

Fly crosses were conducted using standard procedures at 25°C. Pupal developmental ages are expressed as hours after puparium formation (APF) with white pre-pupae defined as 0 hour APF. The following stocks were used: *D-cbl^K26^* and *D-cbl^7^* (this study), *GMR-hid^10^* [62], *ey*-*Flp*; *P[ubi-GFP] FRT80B* (provided by Georg Halder), *UASp-P35* [63], *UAS-EGFR^DN^* [6], *sev-ras^N17^* [64], *sev-yan^act^* [47], *sev^d2^* [53]. To generate *D-cbl* mutant clones, *D-cbl^K26^ FRT80B* and D-*cbl^7 ^FRT80B* flies were crossed to *ey*-*FLP*; *P[ubi-GFP] FRT80B*. Clones are marked by loss of GFP. *GMR-EGFR^DN^* is *GMR-Gal4 UAS-EGFR^DN^*.

### Immunohistochemistry

Eye imaginal discs from the indicated larval or pupal stages were dissected and immunohistochemical labeling was performed as described [65]. The following antibodies were used: rat anti-Elav (1∶60) and rabbit anti-phosphotyrosine antibody (1∶500, both provided by G. Halder); anti-Svp (1∶100, provided by R. Schulz); anti-Rough (1∶50, provided by K. Choi); Rabbit anti-cleaved Caspase-3 (1∶200; Cell Signaling Technology); dp-ERK (1∶2,000; Sigma); anti-Pros (1∶50), mouse anti-Dlg (1∶50) anti-Yan (1∶40), anti-Cut (1∶100) (all DSHB). Fluorescently-conjugated secondary antibodies are from Jackson ImmunoResearch and were used at dilutions of 1∶400. Images were captured using a Olympus Optical FV500 confocal microscope.
